# Forsythoside B ameliorates diabetic cognitive dysfunction by inhibiting hippocampal neuroinflammation and reducing synaptic dysfunction in ovariectomized mice

**DOI:** 10.3389/fnagi.2022.974690

**Published:** 2022-10-28

**Authors:** Xinyu Nan, Qi Sun, Xiaoyu Xu, Ying Yang, Yanfeng Zhen, Yameng Zhang, Haixia Zhou, Hui Fang

**Affiliations:** ^1^Department of Internal Medicine, Hebei Medical University, Shijiazhuang, Hebei, China; ^2^Department of Orthopedics, Hebei General Hospital, Shijiazhuang, Hebei, China; ^3^Tangshan Gongren Hospital, Tangshan, Hebei, China; ^4^Department of Internal Medicine, North China University of Science and Technology, Tangshan, Hebei, China

**Keywords:** diabetes-associated cognitive impairment, ovariectomy, forsythoside b, inflammation, synaptic dysfunction

## Abstract

**Background:**

Diabetes-associated cognitive impairment (DACI) is a common complication of diabetes, and studies have shown that DACI is more severe in postmenopausal patients with diabetes. Forsythoside B (FTS⋅B) can inhibit inflammation and reduce synaptic dysfunction, which can improve cognitive function. However, it has not been confirmed whether FTS⋅B has a reversing or retarding effect on postmenopausal diabetic encephalopathy.

**Methods:**

Seven days after bilateral ovariectomy (OVX) or sham surgery, adult female C57 mice (*n* = 15/group) received intraperitoneal injection of streptozotocin (60 mg/kg/day/L) and citrate buffer for 5 consecutive days to induce diabetes mellitus (DM). Fourteen days later, ovariectomized diabetic mice were given intraperitoneal injection of FTS⋅B (100, 150 mg/kg/day/L) and subcutaneous injection of 17β-estradiol (1 mg/kg) for 8 weeks [OVX + DM + low-FTS⋅B group (L-F), OVX + DM + high-FTS⋅B group (H-F), and OVX + DM + 17β-estradiol (ER)]. In addition, the following control groups were defined: Sham, OVX, DM, and OVX + DM (O + D). Fasting plasma glucose, body weight and blood insulin levels were determined in each group of mice. Next, their cognitive function was tested through behavioral experiments. Hematoxylin & eosin (H&E) and Nissl staining were used to detect the morphological changes in the hippocampus. The aggregation of amyloid beta (Aβ) and the hyperaggregation of p-tau were assessed by immunohistochemistry. Interleukin-1β (IL-1β), interleukin-6 (IL-6), tumor necrosis factor-α (TNF-α), brain-derived neurotrophic factor (BDNF), post-synaptic density-95 (PSD-95), synaptophysin, and synapsin-1 expression in the hippocampus was detected by real-time polymerase chain reaction (RT-PCR) and western blot analysis.

**Results:**

FTS⋅B can decrease fasting glucose and blood insulin level. Behavioral results showed that cognitive decline was the most severe in the O + D group, and the ER, L-F, and H-F groups revised the cognitive decline. Compared to the O + D group, more normal morphology, which has obvious nucleoli and clear nuclear membrane, was observed by H&E and Nissl staining in the ER, L-F, and H-F groups. FTS⋅B alleviated DACI by reducing the aggregation of Aβ and the hyperaggregation of p-tau in the hippocampus. Moreover, the protein and mRNA expression showed that FTS⋅B not only inhibited inflammation by decreasing IL-1β, IL-6, and TNF-α but also modulated synaptic plasticity by increasing BDNF, PSD-95, synaptophysin, and synapsin-1.

**Conclusion:**

These results suggest that FTS⋅B may be a novel therapeutic target for postmenopausal diabetic encephalopathy treatment.

## Introduction

Diabetes is a chronic metabolic disorder that is currently a serious health problem worldwide. A previous study predicted that the number of patients with diabetes may reach 578 million by 2030 and 700 million by 2045 worldwide (Piatkowska-Chmiel et al., 2022). One of the long-term complications of diabetes in the nervous system is called diabetic encephalopathy (DACI), which is characterized by cognitive impairment and neuropathological changes, mainly manifested by altered synaptic plasticity (Wang B. N. et al., 2021), extracellular plaques of aggregated amyloid beta (Aβ), and neurofibrillary tangles composed of hyperphosphorylated tau protein (Pang et al., 2022). With the intensification of the global aging of society, in recent years, the incidence of diabetes mellitus (DM) has been rising, particularly in post-menopausal women (Bragg et al., 2017; Wang B. N. et al., 2021), which brings huge medical and economic burdens to the world and seriously affects people’s daily life and work. Thus, it is necessary to explore the related pathogenesis of menopausal diabetic encephalopathy and to develop an effective drug to treat it.

The inflammatory factors, such as tumor necrosis factor (TNF) and interleukins (IL-1β and IL-6), and free radicals, can lead to cellular damage in the brain, including mitochondrial damage and neuronal apoptosis, which in turn affects cognitive function (Skundric and Lisak, 2003; Chung et al., 2015; Sadeghi et al., 2016). Brain-derived neurotrophic factor (BDNF) is a factor that effectively regulates synaptic plasticity and has a function in promoting learning and memory (Jin et al., 2020). BDNF can modulate synapse-related proteins such as synaptophysin, synapsin-1, and post-synaptic density-95 (PSD-95), which are essential for synaptic plasticity and cognition (Liu et al., 2012; Wang et al., 2016). Therefore, neuroinflammation, neurotrophic factors, and synaptic proteins may be involved in synaptic plasticity damage, which is considered to be the neuropathogenesis of diabetic encephalopathy, to a large extent (Podda et al., 2016).

Forsythoside B (FTS⋅B) is one of the main active components of the air-dried fruit of Forsythia (Wang et al., 2018), and the current study showed that the neuroprotective effect of FTS⋅B depends on multiple antioxidant, anti-apoptotic, anti-inflammatory, and neurogenesis-promoting properties (Jiang et al., 2012). However, whether FTS⋅B can delay menopausal diabetic encephalopathy has not yet been reported and studied. To the best of our knowledge, in this study, we used an ovariectomized diabetic mice model to study the related mechanism of FTS⋅B in the treatment of menopausal diabetic encephalopathy for the first time. We found that FTS⋅B could be a potential drug candidate for the treatment of menopausal diabetic encephalopathy by inhibiting hippocampal neuroinflammation and reducing synaptic dysfunction.

## Materials and methods

### Animals and experimental design

**Scheme 1:**
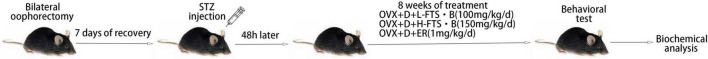
The scheme illustrates the stages of the experiment.

Female C57BL mice (8-weeks-old) were purchased from the Yi Wei Wo Technology Co., Ltd., Shijiazhuang, China. The mice were randomly divided into seven groups as follows: Sham control (Sham); DM; ovariectomy (OVX); ovariectomy and diabetes (O + D); ovariectomy, diabetes, and 17β-estradiol (1 mg/kg) (ER); ovariectomy, diabetes, and low-FTS⋅B (100 mg/kg) (L-F); and ovariectomy, diabetes and high-FTS⋅B (150 mg/kg) (H-F). After anesthesia, each mouse underwent either sham operation (Sham, only a skin incision was made and then sutured) or bilateral ovariectomy. All of the animals were given prophylactic antibiotics (penicillin-G; 20,000 U) beginning soon after surgery and lasting for 3 days. After ovariectomy and 1 week of recovery, 60 mg/kg streptozotocin (freshly prepared, pH 4.5 in citrate buffer) was applied intraperitoneally to induce diabetes. In the control group, ovaries were identified but not removed, and these mice received citrate buffer as a streptozotocin (STZ) vehicle (Wang T. et al., 2021). Forty-eight hours after STZ injections, mice with blood glucose levels above 200 mg/dl were considered diabetic. In addition, the glucose tolerance test was used to convince the success of diabetic model. FTS⋅B and 17β-estradiol were administered intraperitoneally once per day for 8 weeks. Body weight was measured once 2 weeks. After the treatment, the mice were used to evaluate the fasting blood glucose levels and blood insulin levels, and then the mice were subjected to the Morris water maze test, Y maze test, and novel object recognition test (NORT). After the behavioral tests, the animals were anesthetized with 10% chloral hydrate (3.5 ml/kg) and perfused initially with 0.9% saline solution *via* cardiac puncture. For immunofluorescence, hematoxylin & eosin (H&E), and Nissl staining, the animals were perfused with 4% paraformaldehyde (PFA) in 0.1 M phosphate-buffered saline (PBS) following the initial saline solution perfusion. Next, the brain tissues were rapidly removed and post-fixed by immersion in 4% PFA for 24 h. For real-time polymerase chain reaction (RT-PCR) and western blot analysis, the whole hippocampus was removed and snap-frozen at −80°C. All animal experiments were conducted following the national guidelines and the relevant national laws on the protection of animals. The summary of the experiment’s timeline is shown as Scheme 1.

### Morris water maze test

After 8 weeks of treatment with FTS⋅B, a water maze test was performed to detect the cognitive level of the mice. The water maze was divided into four quadrants, with a platform placed in the third quadrant. The maze was filled with water containing milk up to 1.5 cm above the platform at a temperature of 22–24°C, with different shapes and colors attached to the walls of the maze. During the training phase, mice were placed into any of the three quadrants without a platform, and it ended when the mice climbed onto the platform. If a mouse did not find the platform within 60 s, the escape latency was recorded as 60 s, and the mouse was carefully guided into the platform, where it remained for 15 s. The mice were then sun-dried and returned to their cages. During the training period, the mice were tested four times a day on the hidden platform for 5 consecutive days to evaluate their learning ability. On the fifth day of training, the platform was removed and the mice were again placed in the maze to test their spatial memory. In the spatial memory ability test, they were placed in water and allowed to swim for 60 s. An IBM computer (Armonk, NY, USA) equipped with a tracking program was used to record the trajectories of the mice, locate the escape latency of the hidden platform, and record the number of times of crossing the platform (Li et al., 2017).

### Y maze

Y maze spontaneous alternate behavior is a measure of spatial working memory during exploratory activities. The experiment was carried out in a three-arm maze with three arms of equal size, labeled A, B, and C. Each arm was 34 cm long, 8 cm wide, and 14.5 cm high, at an angle of 120° from the other two arms. Each mouse was placed at the end of one arm and allowed to move freely through the maze for 5 min. The arm entry times and the sequence number (e.g., ABABCC) of each mouse were recorded manually. An arm entry was considered complete when the mouse’s hind paw was fully on one arm. The measured parameters included the number of consecutive spontaneous alternate performance (SAP) of mice entering three different arms (i.e., ABC, ACB, BCA, and BAC), the number of mice visiting other arms and returning to the same arm (i.e., ABA, ACA, and BAB), and the number of repeated mice visits to the same arm—returning to the same arm (SAR) (i.e., AA, BB, and CC).

The percentage of SAP was calculated as [(alternating times)/(total arm entry − 2)] × 100.

### Novel object recognition test

**Scheme 2:**
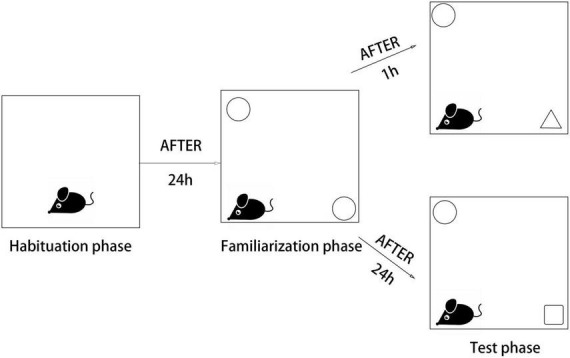
The scheme illustrates the phases of the novel object recognition (NOR) test.

The NORT is a simple behavioral assay of memory that primarily relies on rodent’s innate exploratory behavior in the absence of externally applied rules or reinforcement. The test can be used as a model for the investigation of memory alterations. NORT has habituation, familiarization, and test phases. The experiments were carried out in an isolated chamber, and mice were familiarized with the researcher before the test. The box and objects were cleaned with 70% isopropanol solution before each trial. Habituation phase: the animals were allowed to stay in a box (65 × 45 × 45 cm) for 5 min (Scheme 2). Familiarization phase: the animals were first observed for 5 min in the same box with two identical objects (A + A), and their behavior was recorded with a video camera. Test phase: the animals were observed for 5 min in a box containing a familiar object and a novel object, which was different in shape and color (A + B), and their behavior was recorded with a video camera. Evaluation: results were assigned by comparing the time spent with the familiar and novel objects. The calculation formula is as follows:

Discrimination index (DI) was calculated by dividing the difference in exploration time (seconds) for a familiar object (TF) and a novel object (TN), with the total amount of exploration time for novel and familiar objects: DI = TN/(TN + TF).

### Hematoxylin & eosin and Nissl staining

The brain tissues were carefully collected and fixed in 4% PFA solution for 24 h. Next, they were dehydrated in alcohol and embedded in paraffin. After that, the brain tissue sections were dewaxed, hydrated, and stained with H&E solution (Beyotime, China). For Nissl staining, the slides were stained with cresol violet and Nissl differentiation solution in accordance with the manufacturer’s instructions (Beyotime, China). Finally, the slides were observed under a light microscope (Nikon, Japan).

### Immunofluorescence

The brains were dissected, fixed in PBS with 4% PFA for 2 days, and then frozen into sections. The sections were 10 μm thick and were immunostained with primary Aβ antibody (1:200, Arigobio, China) and tau antibody (1:500, HA, China) at 4°C overnight. The following day, the sections were washed in PBS, incubated with horseradish peroxidase (HRP)–conjugated anti-rabbit secondary antibody at 25°C for 1 h, and incubated with streptavidin-organism HRP complex at 25°C for another 1 h. Next, 5% diaminobenzidine tetrahydrochloride solution and hematoxylin were added as a counterstain (25°C for 5 min). Photomicrographs were obtained using an inverted fluorescence microscope (magnification ×40 and ×200; Nikon, Japan). The intensity of positive staining in the ROI was calculated and defined as the sum of integrated optical density (IOD), and the area of the ROI was also calculated. The average IOD of specific proteins, reported as IOD/mm^2^, was defined as the sum of IOD divided by the area of ROI.

### Real-time polymerase chain reaction

Hippocampal samples were obtained for this analysis. A Gene Amp 7,700 Sequence Detection System (Applied Biosystems, Foster City, CA, USA) and SYBER^®^ Premix Ex Taq™II kit (Takara, Kusatsu, Japan) were used to perform RT-PCR. The primers for the selected genes are listed in Table 1. GAPDH was used as an endogenous control. The changes of relative mRNA transcript levels were reported using the 2 [-Delta C(T)] method as previously described (Mohammadi and Zare, 2020). The experiment was repeated at least three times to ensure accuracy.

**TABLE 1:** Sequences of primers used for RT-PCR.

	Forward primer (5′-3′)	Reverse primers (5′-3′)
GAPDH	GGGGAGCCAAAAGGGTCATCATCT	GAGGGGCCATCCACAGTCTTCT
TNF-α	ACTGAACTTCGGGGTGATCG	CCACTTGGTGGTTTGTGAGTG
IL-1β	TTCGGGCAGGCAGTATCACTCATTG	ACACCAGCAGGTTATCATCATCATCC
IL-6	AGACTTCCATCCAGTTGCCTTCTTG	CATGTGTAATTAAGCCTCCGACTTGTG
PSD-95	TCCGGGAGGTGACCCATTC	TTTCCGGCGCATGACGTAG
synaptophysin	AGACATGGACGTGGTGAATCA	ACTCTCCGTCTTGTTGGCAC
synapsin-1	CCAATCTGCCGAATGGGTACA	GCGTTAGACAGCGACGAGAA
BDNF	TCATACTTCGGTTGCATGAAGG	ACACCTGGGTAGGCCAA

### Western blot analysis

Tissues were collected and dissolved in a radioimmunoprecipitation analysis buffer using a cocktail of protease inhibitors. Protein lysate was electrophoresed with sodium dodecyl sulfate-polyacrylamide gel and transferred to nitrocellulose membranes. Next, 5% bovine serum albumin was used to block the membranes at 4°C for 2 h. The membranes were subsequently incubated with the following primary antibodies at 4°C overnight: BDNF (1:1,000, Abcam), PSD-95 (1:1,000, Abcam), synaptogenin (1:1,000, Abcam), synapsin-1 (1:1,000, Abcam), TNF-α (1:2,000, HA), IL-6 (1:2,000, HA), IL-1β (1:2,000, HA), and the reference protein β action (1:100,000, HA). After washing with TBST, the membranes were incubated with HRP-conjugated secondary antibody at a dilution of 1:2,000 at 4°C for 4 h. An enhanced chemiluminescence kit (Merck Millipore, Billerica, MA, USA) and imaging system (Bio Spectrum 600; UVP, Upland, CA, USA) were used to visualize the bands. ImageJ (National Institutes of Health, Bethesda, MD, USA) was used to quantify the blots.

### Statistical analysis

All data were analyzed using SPSS software (SPSS, Chicago, IL, USA), and the results were expressed as mean ± standard deviation (SD). The Shapiro–Wilk test and the Bartlett’s test were used to analyze the normality of distribution and homogeneity of variance. One-way analysis of variance (ANOVA) and Fisher’s protected least significant difference test were used to determine statistically significant differences between the groups. The results of radiography scores were analyzed using the Kruskal–Wallis test. *P* < 0.05 was considered to indicate statistical significance.

## Results

### Glucose tolerance curve and the effects of forsythoside B on blood glucose, body weight, and blood insulin levels (FPI) in ovariectomized diabetic mice were analyzed

**FIGURE 1:**
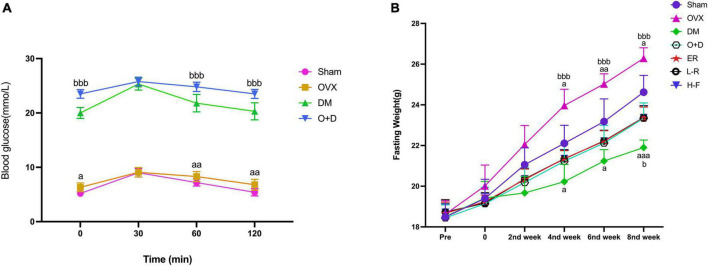
Changes of blood glucose and fasting weight. **(A)** Blood glucose and **(B)** fasting weight in Sham, OVX, DM, O+D, ER, L-F, and H-F groups. Before operation (Pre); after operation and streptozotocin injection (0), while FTS•B and 17β-estradiol were administered from the first week to the eighth week on an alternate day. Compared to Sham group, ^a^*P* < 0.05, ^aa^*P* < 0.01, and ^aaa^*P* < 0.001. Compared to O+D group, ^b^*P* < 0.05, ^bb^*P* < 0.01, ^bbb^*P* < 0.001, and ns indicates not significant.

We detected the levels of fasting blood glucose and blood insulin levels in each group (Table 2). As shown in Figure 1A, the blood glucose levels in the Sham group at 0, 60, and 120 min were significantly lower than those in the OVX group, and the blood glucose levels in the DM group at 0, 60, and 120 min were significantly lower than those in the O + D group. But there was no significant difference between the groups at 30 min. Among them, the DM group showed the lowest level. This suggests that the diabetes model was established successfully. As shown in Table 2, compared with the Sham group, the levels of fasting blood glucose in the OVX, DM, and O + D groups were higher. However, treatment with FTS⋅B and 17β-estradiol significantly reversed this trend. Figure 1B demonstrates that campared with Sham group, the fasting weight was significantly reduced in the DM group and significantly increased in the OVX group. Figure 1B also showed that when compared with the O + D group, the fasting weight in the OVX group was significantly increased in the fourth, sixth, and eighth weeks and in the DM group was significantly reduced in the eighth week. However, no significant differences were found between the treatment groups that received different doses of FTS⋅B and 17β-estradiol. Compared to the Sham group, the FPI level was significantly reduced in the DM and O + D groups (Table 2). After treatment, significant improvements were observed in the ER, L-F, and H-F groups. This suggests that FTS⋅B has anti-diabetic effects.

**TABLE 2:** Effects of forsythoside B on blood glucose and blood insulin levels in ovariectomized diabetic mice.

Group	Blood glucose (mmol/L)	Blood insulin levels (μIU/ml)
Sham	5.21 ± 0.16	40.85 ± 1.14
OVX	6.22 ± 0.42***	39.68 ± 2.48
DM	19.97 ± 1.18***	25.95 ± 0.79***
O + D	24.03 ± 1.04***	24.22 ± 1.42***
ER	11.13 ± 0.33^###^	33.49 ± 1.17^###^
L-F	10.7 ± 1.17^###^	32.79 ± 1.07^###^
H-F	8.2 ± 0.67^###^	34.27 ± 0.69^###^

Compared to Sham, ***P < 0.001. Compared to O + D, ^###^*P* < 0.001. Data are expressed as mean ± SD (*n* = 10).

### Forsythoside B ameliorates cognitive decline of ovariectomized diabetic mice

**FIGURE 2:**
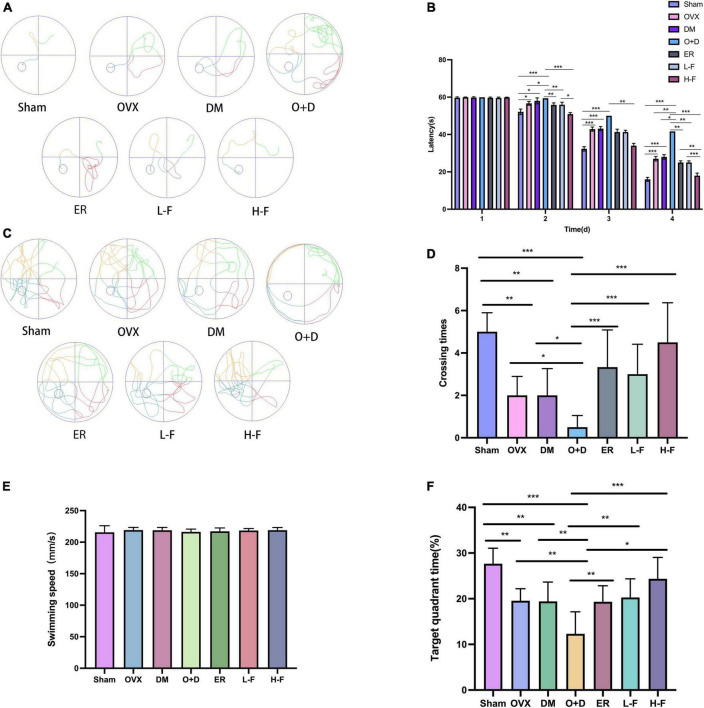
FTS•B significantly improves the learning and memory ability of ovariectomized diabetic mice. Behavioral tests and quantitative analyses of C57 mice. **(A)** Representative tracing graphs showing the training trials. **(B)** Escape latency during the platform trials. **(C)** Representative tracing graphs of the probe trials. **(D)** Number of crossings over the original platform location in the probe trials. **(E)** The swimming speed of each group. **(F)** The target quadrant time. *n* = 6. *P < 0.05, **P < 0.01, ***P < 0.001, and ns indicates not significant.

We used water mazes to evaluate learning and memory in mice. Compared with the Sham group, the OVX, DM, and O + D groups showed more chaotic movements (Figure 2A) and longer escape latency (Figure 2B), and the number of platform crossings decreased (Figures 2C,D). Among them, the O + D group showed the most obvious changes. However, treatment with FTS⋅B and 17β-estradiol significantly reversed this trend, especially in the H-F group (Figures 2A–D). And there was no significant difference between the groups of swimming speed (Figure 2E). Compared with the Sham group, the target quadrant time was significantly reduced in the OVX, DM, and O + D groups. Among them, the O + D group was the most obvious. However, treatment with FTS⋅B and 17β-estradiol significantly reversed this trend, especially in the H-F group (Figure 2F).

**FIGURE 3:**
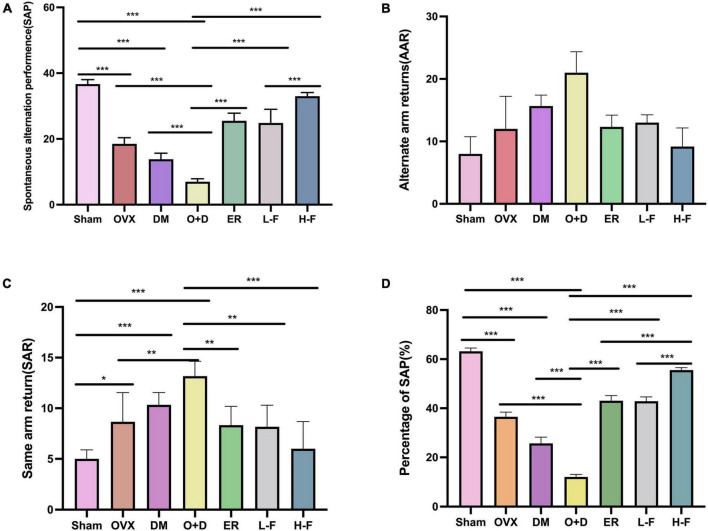
FTS•B significantly improves workspace memory impairment in ovariectomized diabetic mice in the Y-maze test. **(A)** Spontaneous alteration performance (SAP) was defined when an animal visited three different arms (i.e., A, B, and C) consecutively (i.e., ABC, ACB, BCA, or BAC). **(B)** Alternate arm returns (AAR) was defined when an animal visited other arms and then returned to the initial arm (i.e., ABA, ACA, and BAB). **(C)** Same arm returns (SAR) was defined as repeated visits of the same arm (i.e., AA, BB, and CC). **(D)** The percentage of SAP (%); *n* = 6. *P < 0.05, **P < 0.01, ***P < 0.001, and ns indicates not significant.

We assessed working and spatial memory performance by recording spontaneous change behavior in the Y-maze test. As shown in Figure 3, there were significant differences in spatial memory performance among different groups (*P* < 0.05; Figures 3A–C). Compared with the Sham group, the groups OVX, DM, and O + D showed fewer spontaneous alternating movements (Figure 3A). Among them, the O + D group had the fewest spontaneous alternating movements. However, treatment with FTS⋅B and 17β-estradiol effectively increased the percentage of spontaneous changes (Figure 3D).

**FIGURE 4:**
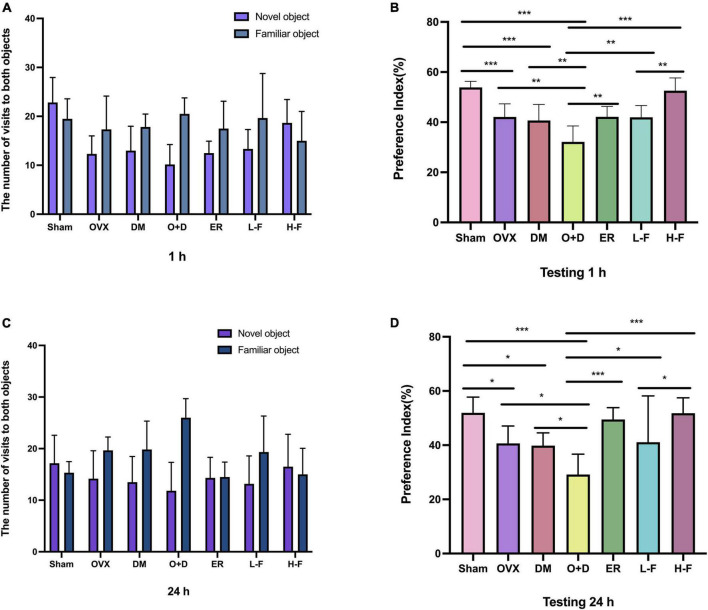
FTS•B can significantly reduce memory impairment in ovariectomized diabetic mice, as indicated by the new object recognition test. The recognition memory was measured by the novel object recognition test where the mice were allowed to explore the familiar and novel objects for 5 min. **(A,C)** The bars show the number of times the different groups of mice visited the two objects during the test phase. **(B,D)** The bar chart shows the preference index (%) for each group. *n* = 6. *P < 0.05, **P < 0.01, ***P < 0.001, and ns indicates not significant.

To confirm the effect of FTS⋅B on the menopausal diabetic encephalopathy, a NORT was also performed. The statistical analysis revealed that the groups OVX, DM, and O + D showed a significant reduction in their interest of the objects in the testing stage, especially of a new one, compared to the animals in the Sham group. However, treatment with FTS⋅B and 17β-estradiol significantly reversed this trend (Figures 4A–D).

Taken together, these data suggest that FTS⋅B treatment significantly improves learning and cognitive impairment associated with menopausal diabetes.

### Forsythoside B reduces hippocampal morphological changes and synaptic dysfunction in ovariectomized diabetic mice

**FIGURE 5:**
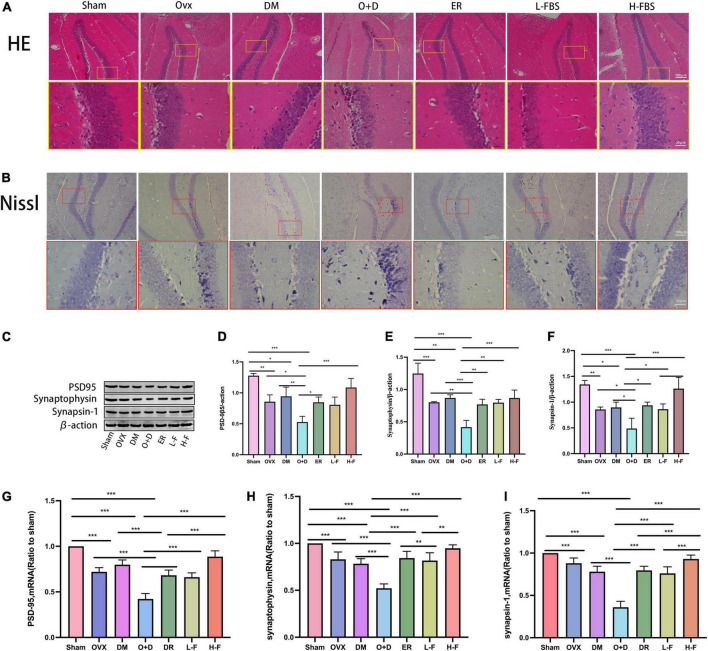
FTS•B reduces hippocampal morphologic changes and synaptic dysfunction in ovariectomized diabetic mice. **(A,B)** Representative hematoxylin and eosin (H&E) and Nissl-stained sections of the hippocampus. **(C)** Representative western blotting showing PSD95, synaptophysin, and synapsin-1 expression levels. **(D–F)** Expression levels of PSD95, synaptophysin, and synapsin-1 were quantitatively analyzed. *n* = 3. **(G–I)** Quantitative analysis of the mRNA expression of PSD95, synaptophysin, and synapsin-1. *n* = 6. *P < 0.05, **P < 0.01, ***P < 0.001, and ns indicates not significant. Scale bars = 100 and 20 μm as indicated.

Next, we evaluated whether FTS⋅B treatment affected hippocampal morphology in each group of mice. We found that the hippocampal region of the Sham group showed normal morphology, with obvious nucleoli and clear nuclear membrane (Figures 5A,B). However, in the OVX, DM, and O + D groups, hippocampal neurons were lost and hippocampal cells were sparsely arranged with blurred boundaries. The O + D group was the most severely affected. Importantly, FTS⋅B and 17β-estradiol therapy largely reversed these abnormalities, particularly in the H-F group (Figures 5A,B). These results suggest that FTS⋅B has a significant neuroprotective effect, as it inhibits hippocampal neuron loss and structural changes.

We also assessed the expression levels of synaptic associated proteins (PSD-95, synaptogenin, and synapsin-1) in the hippocampus, which form the molecular basis of synaptic plasticity. PSD-95 (Figures 5C,D), synaptogenin (Figures 5C,E), and synapsin-1 (Figures 5C,F) proteomic levels were significantly decreased in the OVX, DM, and O + D groups compared with the Sham group. Among them, the decrease was most obvious in the O + D group. After treatment with FTS⋅B and 17β-estradiol, the expression of these three protein groups was significantly increased in ovariectomized diabetic mice, especially in the H-F group (Figures 5C–F). RT-PCR results were consistent with the western blot results (Figures 5G–I). These results suggest that FTS⋅B enhances the expression level of synaptic associated proteins in the hippocampus, which may indicate the dose-dependent effect of FTS⋅B *in vivo*.

### Forsythoside B inhibited the aggregation of amyloid beta and the hyperaggregation of p-tau in the hippocampus and increased the level of brain-derived neurotrophic factor in the hippocampus of ovariectomized diabetic mice

**FIGURE 6:**
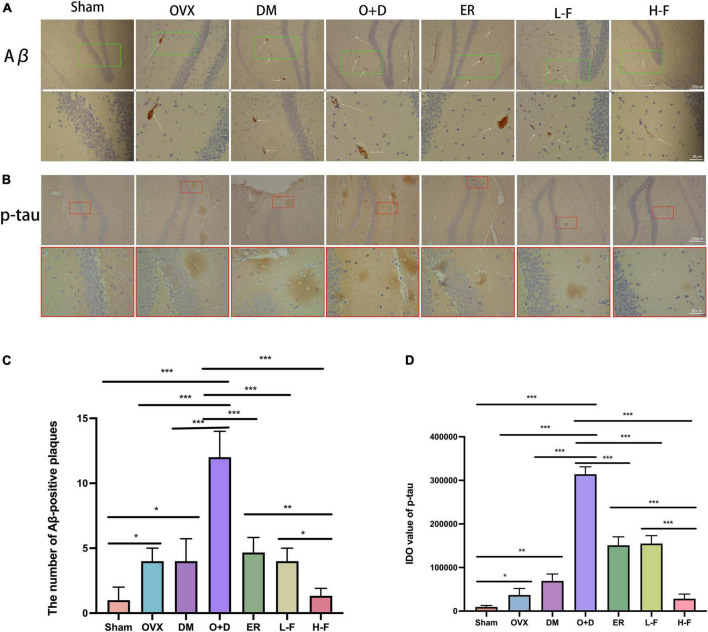
FTS•B treatment inhibits the accumulation of Aβ and *p*-tau in the hippocampus of ovariectomized diabetic mice. **(A,B)** Immunohistochemistry assay for Aβ and *p*-tau in the hippocampus in different groups. The thin white arrow indicates Aβ-positive plaques. **(C,D)** Quantitative analysis of Aβ and *p*-tau expression. *n* = 3. *P < 0.05, **P < 0.01, ***P < 0.001, and ns indicates not significant. Scale bars = 100 and 20 μm as indicated.

The immunohistochemical results showed that the number of Aβ-positive plaques in the hippocampus of the OVX, DM, and O + D groups was significantly higher than that of the Sham group, and the difference was the most significant in the O + D group. However, FTS⋅B and 17β-estradiol administration significantly reduced the deposition of Aβ in the O + D group, especially at group of H-F (Figures 6A,C). As a microtubule-associated protein, tau stabilizes the neuronal cytoskeleton, and its high phosphorylation may lead to the formation of toxic neurofibrillary tangles in diseases such as diabetic encephalopathy and AD (Yan et al., 2018). In this study, hyperaggregation of p-tau was found in the hippocampus of mice in the OVX, DM, and O + D groups compared with the Sham group. The hyperaggregation of p-tau was most severe in the O + D group. These differences were significantly reduced after FTS⋅B and 17β-estradiol treatment, especially in the H-F group (Figures 6B,D).

**FIGURE 7:**
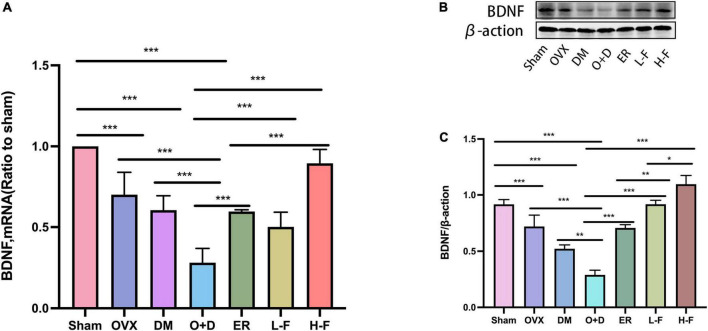
FTS•B increases the level of BDNF in the hippocampus of ovariectomized diabetic mice. **(A)** Quantitative analysis of the mRNA expression of BDNF. *n* = 6. **(B)** Representative western blotting showing BDNF expression level. **(C)** Quantitative analysis of BDNF expression. *n* = 3. *P < 0.05, **P < 0.01, ***P < 0.001, and ns indicates not significant.

Previous studies have found that BDNF is a neurotrophic factor that can promote the development of immature neurons and increase the survival rate of neurons and synaptic plasticity (Tang et al., 2019). We investigated the effect of FTS⋅B treatment on hippocampal BDNF levels. Our RT-PCR results showed that compared with the Sham group, the mRNA expression levels of BDNF in the hippocampus of the OVX, DM, and O + D groups were significantly reduced, with the lowest mRNA level in the O + D group. However, treatment with FTS⋅B and 17β-estradiol reversed this reduction. In the H-F group was more effective (Figure 7A). Western blot results were consistent with RT-PCR results (Figures 7B,C). These data suggest that FTS⋅B treatment can inhibit the deposition of Aβ and hyperaggregation of p-tau and increase the expression level of BDNF in ovariectomized diabetic mice.

### Forsythoside B mediates regulation of neuroinflammation-related protein expression and activation in the hippocampus of ovariectomized diabetic mice

**FIGURE 8:**
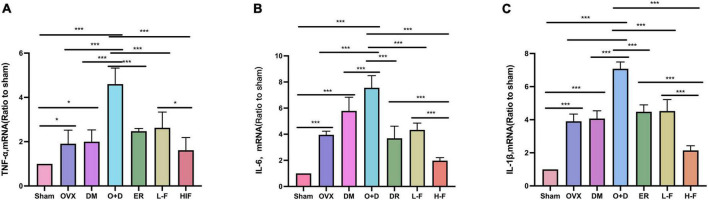
FTS•B inhibits the mRNA expression of factors related to hippocampal neuroinflammation in ovariectomized diabetic mice. **(A–C)** Quantitative analysis of the mRNA expression of TNF-α, IL-6, and IL-1β in the hippocampus. *n* = 6. *P < 0.05, **P < 0.01, ***P < 0.001, and ns indicates not significant.

**FIGURE 9:**
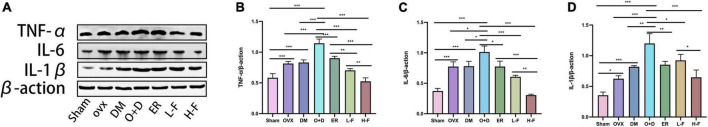
FTS•B inhibits the expression of factors related to hippocampal neuroinflammation in ovariectomized diabetic mice. **(A)** Representative western blotting showing TNF-α, IL-6, and IL-1β expression levels in the hippocampus. **(B–D)** Quantitative analysis of TNF-α, IL-6, and IL-1β expression. *n* = 3. *P < 0.05, **P < 0.01, ***P < 0.001, and ns indicates not significant.

We investigated whether neuroinflammation exists in patients with postmenopausal diabetes and whether FTS⋅B can prevent neuroinflammation. We assessed the expression levels of TNF-α, IL-6 and IL-1β in the hippocampus using RT-PCR and western blot. RT-PCR results showed that compared with the Sham group, the expression levels of TNF-α, IL-6, and IL-1β in the hippocampus of the OVX, DM, and O + D groups were significantly increased, and the increase was most significant in the O + D group. FTS⋅B and 17β-estradiol significantly reduced the mRNA expression levels of these factors, especially in the H-F group (*P* < 0.05, Figures 8A–C). Western blot results were consistent with the RT-PCR results (*P* < 0.05, Figures 9A–D). These results suggest that FTS⋅B inhibits neuroinflammation in the hippocampus of ovariectomized diabetic mice.

## Discussion

Diabetes is a chronic metabolic disease that can cause structural and functional changes in the central nervous system, leading to cognitive decline (Liu et al., 2020). Previous studies have shown that estrogen deficiency can affect the cognitive function (Albert and Newhouse, 2019). Estrogen plays important roles in the brain regions associated with learning and memory, such as the hippocampus and frontal cortex. These include neurotrophic and neuroprotective effects, increased long-term potentiation (LTP) and dendritic spine density, and upregulation of BDNF and growth receptors (Bansal and Chopra, 2015; Goyal and Garabadu, 2020). There is a synergistic effect between estrogen deficiency and diabetes, which can significantly aggravate the symptoms of cognitive impairment in postmenopausal diabetic patients. Therefore, it is very important to explore the molecular mechanisms behind cognitive impairment in postmenopausal diabetic patients and to identify effective treatment strategies. However, there have been few studies on menopausal diabetic encephalopathy. Therefore, in this study, we performed bilateral ovariectomy in C57 mice and administered intraperitoneal injection of STZ after surgery to simulate a postmenopausal diabetic mouse model. We showed that postmenopausal diabetic mice developed cognitive impairment and impaired learning and memory functions. We also found that FTS⋅B could alleviate the symptoms and pathological changes of cognitive dysfunction in the mice by inhibiting hippocampal neuroinflammation and reducing synaptic dysfunction. This study shows that FTS⋅B can effectively inhibit neuroinflammation of the central nervous system and delay the progression of menopausal diabetic encephalopathy. Thus, FTS⋅B is a potential therapeutic drug for menopausal diabetic encephalopathy.

Forsythoside B, a phenylethanoid glycoside, is a traditional oriental medicine. Some studies have shown that FTS⋅B can inhibit inflammation, suppress oxidative stress, and promote blood circulation (Jiang et al., 2010b). Jiang et al. (2010a) found that FTS⋅B played a significant neuroprotective role in an SD ischemia–reperfusion rat model by inhibiting inflammation. Qu et al. (2021) showed that synaptic plasticity is closely associated with cognitive function. However, it has not been reported whether FTS⋅B can improve cognitive impairment in menopausal diabetic mice. In this study, we found that FTS⋅B could reduce cognitive impairment in menopausal diabetic mice, inhibit inflammatory factors, and reduce synaptic dysfunction, which effectively improve their cognitive function.

A large number of studies have shown that DACI can cause changes in animal behavior; so, behavioral experiments can be used as an important detection method in the study of diabetic encephalopathy (Yan et al., 2018; Tang et al., 2019; Mohammadi and Zare, 2020; Piatkowska-Chmiel et al., 2022). Piatkowska-Chmiel et al. administered fructose solution + STZ intraperitoneal injection for 4 weeks to induce diabetes in CD-1 mice. Water maze, Y maze, and new object recognition experiment were applied to detect the model group mice. The results showed significant cognitive impairment in the model group. After the treatment with newly synthesized adamantane derivatives and dipeptidyl peptidase 4, the behavioral performance significantly improved (Piatkowska-Chmiel et al., 2022). In addition, it was found by behavioral testing that the learning and memory ability of the high-glucose and high-fat combined with STZ intraperitoneal injection mouse model group was significantly lower compared with the control group. However, after treatment with dihydromyricetin, the learning and memory ability was significantly improved, indicating that dihydromyricetin can significantly improve cognitive dysfunction in type 2 diabetic mice (Zhu et al., 2017). In this study, the behavioral test results were basically consistent with the abovementioned studies. We found that compared with the control group, the postmenopausal diabetic mouse model group exhibited more chaotic movements and longer escape latency in the water maze test, and the number of cross-platforms in the water maze was reduced. In the Y-maze experiment, the spontaneous alternating movements were reduced in the model group. In the novel object recognition experiment, the mice in the model group were significantly less interested in objects in the experimental stage, especially in novel objects. However, FTS⋅B treatment significantly reversed the above phenomenon, indicating that FTS⋅B can effectively improve cognitive dysfunction in postmenopausal diabetic mice.

The hippocampus is an important functional region of the brain, and it is mainly involved in memory, learning, executive ability, and attention regulation (Wikenheiser and Schoenbaum, 2016). Arroyo-Garcia et al. (2021) showed that the morphological changes in the hippocampus are closely related to a decline in cognitive function. In addition, some studies used H&E staining to observe the histological changes in the neurons of the hippocampus, which can be used to assess cognitive function (Yang et al., 2022). In this study, we used H&E staining to observe the changes in hippocampal morphology. Fortunately, the results are consistent with other studies. We also used Nissl staining and found that FTS⋅B significantly improved the hippocampal structure and neuron loss in the ovariectomized diabetic mice model, suggesting that FTS⋅B can significantly improve the cognitive function of menopausal diabetic mice.

Previous studies have not fully elucidated the specific pathogenesis of cognitive impairment in postmenopausal diabetes. It has been reported that Aβ deposition and neurofibrillary tangles are the main pathological changes in brain cognitive impairment (Zhang et al., 2022). Neurofibrillary tangles consist of intracellular self-assembled p-tau bundles that are induced by Aβ deposition and lead to neuronal degeneration (Kim et al., 2015). Aberrant neurogenesis is considered an important causative event of cognitive impairment (Anacker and Hen, 2017; Cuartero et al., 2019). Neurotrophic factors play a crucial role in neurogenesis (Agrimi et al., 2019). It has been found that BDNF is an important neurotrophic factor and its signaling inhibits autophagy in an adult brain by transcriptionally downregulating key (Patterson, 2015; Chou et al., 2020) components of the autophagy machinery and that genetic ablation of BDNF in the nervous system leads to uncontrolled increases in autophagy in the adult brain and is associated with severe synaptic defect (Nikoletopoulou et al., 2017). Decreased levels of BDNF in the hippocampus can aggravate cognitive impairment. In this study, we found that FTS⋅B significantly enhanced the learning and memory abilities of postmenopausal diabetic mice and significantly inhibited the deposition of Aβ and neurofibrillary tangles composed of p-tau protein. However, FTS⋅B significantly increased the expression of BDNF in the hippocampus and effectively improved its cognitive dysfunction.

Neuroinflammation is an important phenotype and a key mediator of DACI and plays an important role in its pathogenesis (Albazal et al., 2021). Neuroinflammation can amplify itself by increasing tauopathy and Aβ deposition through inflammatory cytokines such as IL-1β, IL-6, and TNF-α (Cavanagh and Wong, 2018). Choi et al. (2019) found that the central nervous system inflammation is closely associated with pathological neurodegenerative diseases. Some studies have shown that proinflammatory cytokines (IL-1β, IL-6, and TNF-α) can interfere with insulin signal transduction and participate in insulin resistance and DACI (Degirmenci et al., 2019). However, the relationship between neuroinflammation and menopausal diabetic encephalopathy is unknown. In this study, we found that FTS⋅B significantly improved cognitive dysfunction by reducing the expression of inflammatory cytokines IL-1β, IL-6, and TNF-α in the hippocampus of postmenopausal diabetic mice.

In this study, we used bilateral ovariectomy and STZ intraperitoneal injection to create a menopausal diabetes model. FTS⋅B was found to effectively inhibit the progression of menopausal diabetic encephalopathy through behavioral experiments, histological staining, and mRNA and protein expression levels of inflammation. However, there are some limitations in this study. The specific targets and related mechanisms of FTS⋅B in the hippocampus have not been fully understood. Furthermore, the subtle morphological changes of synapses were not studied in this experiment. In future studies, we will use a staining technique, such as Golgi staining, to visualize changes in the synaptic structure. Meanwhile, we will focus on *in vitro* experiments and further explore its deeper mechanism by applying anti-inflammatory pathway inhibitors.

## Conclusion

**FIGURE 10:**
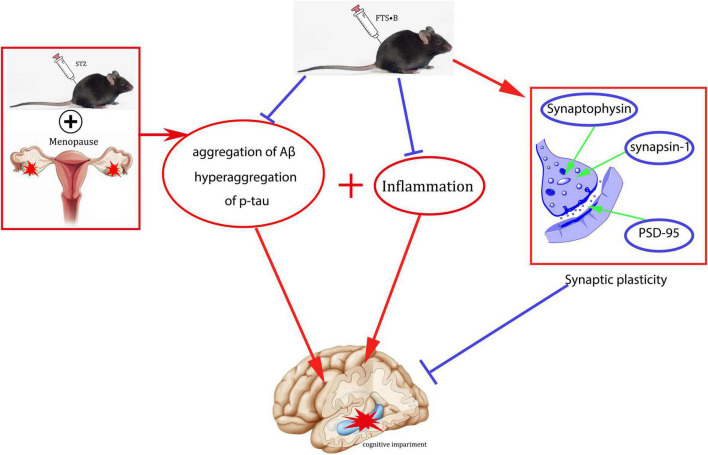
Schematic diagram of a potential mechanism of FTS•B in the treatment of cognitive dysfunction in ovariectomized diabetic mice.

In conclusion, this study systematically reports the neuroprotective effects of FTS⋅B in ovariectomized diabetic mice, including decreased fasting blood glucose, improved behavioral performance, normalized hippocampal morphology, increased synaptic plasticity, and improved Aβ deposition and tau phosphorylation. FTS⋅B can also increase the expression of BDNF and inhibit the expression of inflammatory factors (Figure 10), thereby restoring the cognitive function of postmenopausal diabetes. Therefore, this study suggests that FTS⋅B may be a potential treatment for postmenopausal diabetic encephalopathy.

## Data availability statement

The raw data supporting the conclusions of this article will be made available by the authors, without undue reservation.

## Ethics statement

The animal study was reviewed and approved by the North China University of Science and Technology Application.

## Author contributions

HF designed the study. XN and QS kept the animal and analyzed the data. XX, YY, YFZ, YMZ, and HZ critically reviewed the data. XN drafted the manuscript. All authors contributed to interpreting the data, critically revising the manuscript, and approved the final version.
